# Prophylactic laparoscopic total gastrectomy in a patient with situs inversus totalis

**DOI:** 10.1093/jscr/rjaa475

**Published:** 2021-05-15

**Authors:** Jonathan Sivakumar, Gary Crosthwaite

**Affiliations:** Clinical Institute General Surgery and Gastroenterology, Epworth Healthcare, Richmond, Victoria, Australia; Department of Surgery, The University of Melbourne, Melbourne, Victoria, Australia; Clinical Institute General Surgery and Gastroenterology, Epworth Healthcare, Richmond, Victoria, Australia; Department of Surgery, The University of Melbourne, Melbourne, Victoria, Australia

**Keywords:** situs inversus, gastrectomy

## Abstract

A rare case was demonstrated whereby a prophylactic laparoscopic total gastrectomy was performed in a 29-year-old male with a CDH1 gene mutation in the context of rare anatomical anomaly, situs inversus totalis (SIT).

This report provides radiological and laparoscopic images detailing the patient’s unique anatomy. We also describe the operative approach and technical challenges whilst accounting for the patient’s anatomical anomaly.

This is the first known case of a laparoscopic total gastrectomy for a CDH1 gene mutation in a patient with SIT. With sufficient pre-operative evaluation and meticulous intra-operative caution, this technically complex operation is safe and feasible in patients with mirror-image anatomy as seen in SIT.

## INTRODUCTION

Hereditary diffuse gastric cancer is a rare autosomal dominant syndrome that is mostly due to germline mutations in the CDH1 gene [1]. These mutations confers at least a 70% lifetime risk of developing gastric cancer, and a prophylactic total gastrectomy is recommended prior to the onset of symptoms [2]. The approach of this procedure laparoscopically has been previously described [3]. We report a unique case of performing a prophylactic laparoscopic total gastrectomy for a patient with a CDH1 gene mutation in the context of rare anatomical anomaly, situs inversus totalis (SIT). While the procedure is already technically complex, this is further compounded when considering the structural variations in this patient.

## CASE REPORT

**Figure 1:**
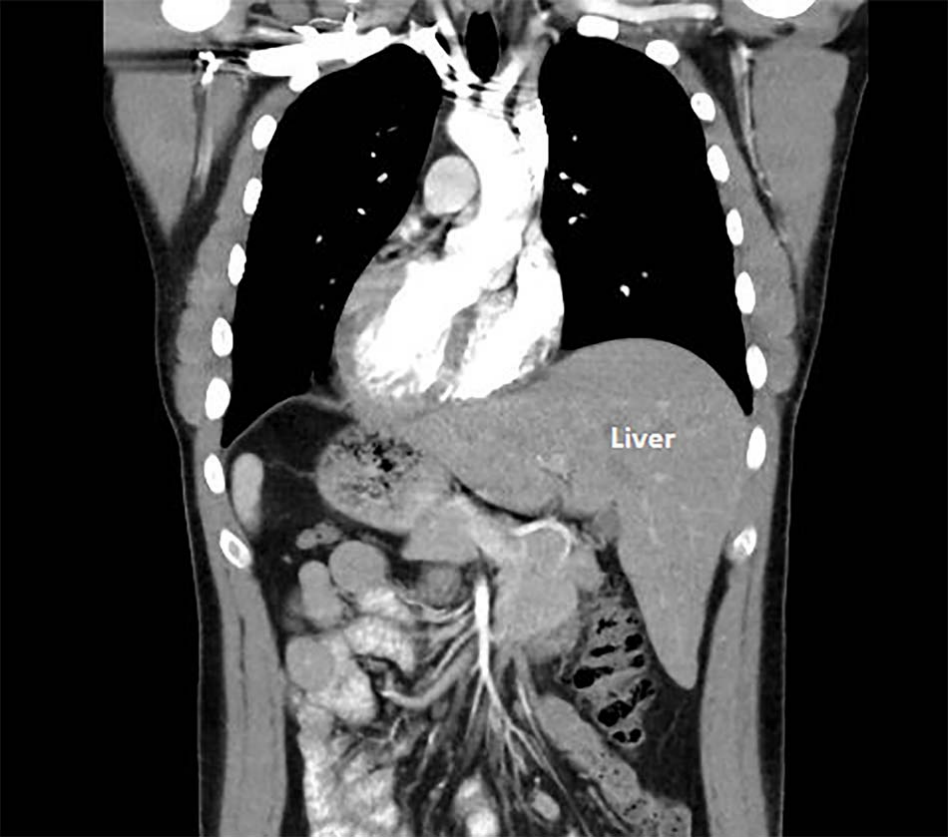
Coronal cross-section image of computed tomography scan.

A 29-year-old male was referred for consideration of surgery in the setting of a CDH1 gene mutation. After thorough counseling and after observing the reassuring outcomes of his own family members that had undergone this surgery for the same genetic mutation, he elected to undergo a laparoscopic total gastrectomy and he elected to undergo a laparoscopic total gastrectomy. A computed tomography (CT) scan was undertaken for operative planning and demonstrated mirror-image anatomy, a characteristic of SIT (Fig. 1).

**Figure 2:**
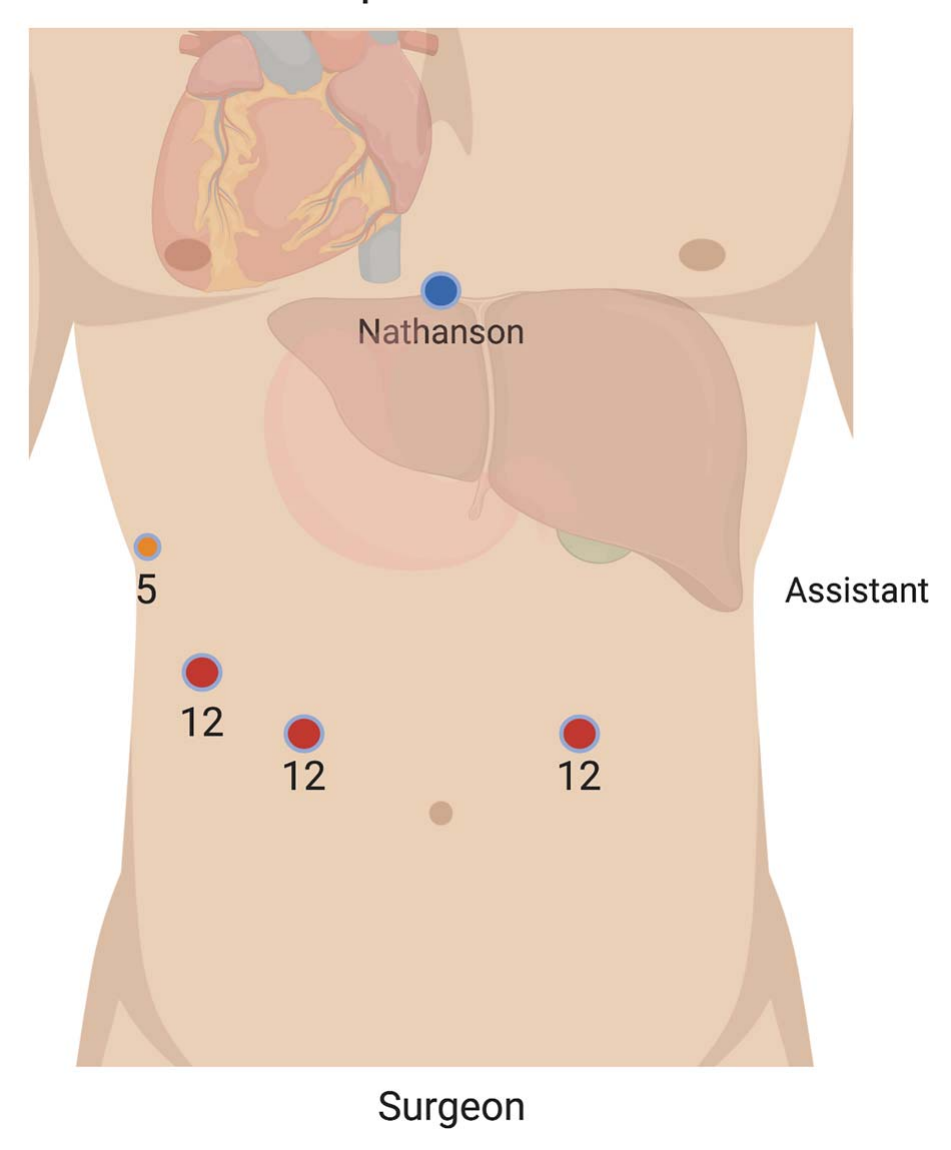
Abdominal port placement

**Figure 3:**
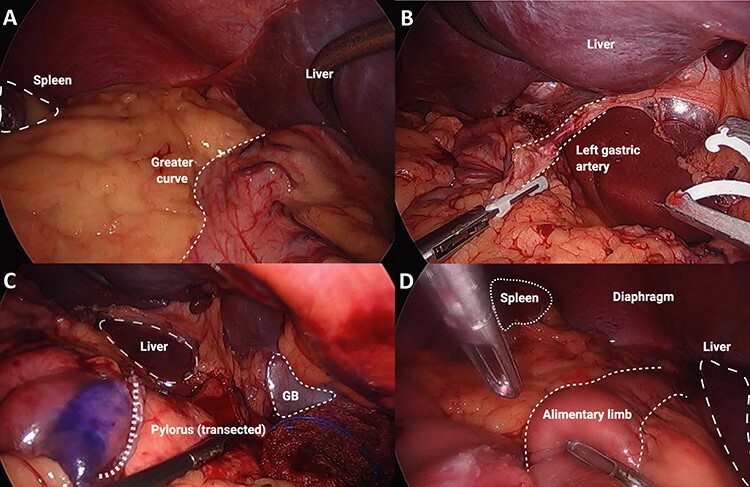
Series of intra-operative images: (**A**) major abdominal structures noted on opposite sides, in keeping with situs inversus totalis, (**B**) left gastric artery demonstrated coursing to the patient’s right side, (**C**) transected pylorus course from the patient’s right side, (**D**) alimentary limb shown after completion of oesophago-jejunal anastomosis.

This procedure was performed with the patient in French position, where the primary surgeon was positioned between the patient’s legs and the assistant on the patient’s right, diametrically opposite to the standard layout for a gastrectomy. Access was obtained via optical entry in the lower aspect of the left upper quadrant. A 12-mm port was then inserted in the lower part of the right upper quadrant mirroring the first port; a 5-mm port was inserted at the lateral right costal margin and a 12-mm port was inserted between these two. A Nathanson liver retractor was used via a subxiphoid incision (Fig. 2). This configuration was to account for the anatomical variations of SIT. A ‘reversed abdomen’ in the sagittal plane was noticed on entry (Fig. 3). Key steps involved in carrying out this procedure include an end-to-side oesophago-jejunostomy with the use of an EEA™ anvil-stapler inserted through a transverse oesophagotomy and a side-to-side jejuno-jeunostomy Roux-en-Y reconstruction where the alimentary limb was 55 cm in length. The operative time was 392 minutes.

This patient’s recovery was uneventful, diet was commenced within 48 hours, and he was discharged six days post-operatively. Although the pre-operative gastroscopy was macroscopically and microscopically unremarkable, the histopathological assessment of the operative specimen demonstrated grade 3 poorly cohesive signet ring cells present within the gastric fundus and no metastases to lymph nodes (pT1aN0M0, Stage IA according to American Joint Committee on Cancer, 8^th^ edition). This patient will continue to be followed up for three months in the first year, six months in the second year and annually thereafter, with a focus on surveying nutritional status.

## DISCUSSION

This patient’s case presents several unique clinical issues. The co-existence of CDH1 disease and SIT is a rare phenomenon that has not been previously reported in the literature. SIT is a rare autosomal-recessive congenital anomaly in which thoracic and abdominal structures are transposed to the opposite side of the body. It carries an incidence of one in every 8000 births [4]. While the treatment is no different to standard, the anatomical difference may pose a distinct challenge intra-operatively.

SIT is commonly associated with vascular aberrations, and previous relevant surgical cases have strongly recommended pre-operative anatomical arterial mapping [5]. We sought a CT angiography pre-operatively to ascertain the anatomy and detect any abnormal vascular structures that may complicate or confuse the surgery. Aside from the complete transposition of vascular structures to the opposite side, there was no additional variation to the branching pattern in the abdomen (Fig. 3).

It is necessary for the surgical team to cautiously arrange the operating theatre in such a way that accounts for SIT. This ensures the procedure is carried out safely and allows the surgeon to operate in a more ergonomic fashion. In the present case, both port placement and operator positions were reversed to accommodate for the mirror-image anatomy. This also required the surgeon to be relatively ambidextrous in using laparoscopic instruments. Given the challenging orientation and the high risk of iatrogenic injury from unintended tissue handling, it was important to continually reassess the operative setting whilst being conscious of the patient’s anatomy. This was a major factor contributing to the prolonged operative time compared to the conventional case.

To our knowledge, this is the third published case of a laparoscopic total gastrectomy being performed in the setting of SIT [6, 7], and the first case to be performed prophylactically for a patient with a CDH1 germline mutation. These similar studies also note the technical challenges and increased operating time of the case because of the slow adaption to the mirror image anatomy.

With sufficient pre-operative evaluation and meticulous intra-operative caution, a laparoscopic total gastrectomy is safe and feasible in patients with SIT.

## DECLARATIONS

Ethics approval and consent to participate: This case report was approved by the ethics committee of Research Development and Governance Unit at Epworth Healthcare. Written informed consent was obtained from the patient to publish this case report.

## CONFLICT OF INTEREST STATEMENT

None declared.

## FUNDING

None.
